# Quantifying the germination response of *Parthenium hysterophorus* at various temperatures and water potentials by using population-based threshold model

**DOI:** 10.3389/fpls.2022.961378

**Published:** 2022-08-10

**Authors:** Irfan Afzal, Muhammad Akram, Talha Javed, Faryal Ali, Hazem M. Kalaji, Jacek Wróbel, Arkadiusz Telesiński, Jacek Mojski, Mohamed A. A. Ahmed

**Affiliations:** ^1^Seed Physiology Laboratory, Department of Agronomy, University of Agriculture, Faisalabad, Pakistan; ^2^Department of Plant Physiology, Institute of Biology, Warsaw University of Life Sciences SGGW, Warsaw, Poland; ^3^Institute of Technology and Life Sciences—National Research Institute, Raszyn, Poland; ^4^Department of Bioengineering, West Pomeranian University of Technology in Szczecin, Szczecin, Poland; ^5^Twój Swiat Jacek Mojski, Lukow, Poland; ^6^Fundacja Zielona Infrastruktura, Lukow, Poland; ^7^Plant Production Department (Horticulture - Medicinal and Aromatic Plants), Faculty of Agriculture (Saba Basha), Alexandria University, Alexandria, Egypt

**Keywords:** parthenium, climate change, hydrotime, thermal time, weed management

## Abstract

Predicting the germination behavior of parthenium weed against different conditions of temperature and osmotic stress is helpful for studying the growth and development history of parthenium in different ecological contexts. Sustainable weed control strategies based on population-based threshold (PBT) models are profitable tools for crop planting date, herbicide application, and tillage operation time. To predict the emergence of parthenium by using thermal time (TT), hydrotime (HT), and hydrothermal time (HTT) analyses, seeds were exposed to varying constant temperatures (5, 10, 15, 20, 25, 30, 35, and 40°C) and water potentials (− 0.25, − 0.5, − 0.75, and − 1.0 MPa) under a controlled environment. Parthenium seeds showed better responses in terms of higher germination percentage and lower germination time at 20 and 25°C. The use of the germination modeling approach proposed the base temperature (7.2°C), optimum temperature (20°C), and ceiling temperature (42.8°C) for this weed. Moreover, germination behavior was also studied at different water potentials under different temperature regimes (10, 20, and 30°C). The HTT model predicted higher germination percentages (82.8 and 54.8%) of parthenium seeds at water potentials from 0 to −0.25 MPa, respectively, under a temperature of 20°C, and also identified a base water potential (Ψb_(50_) of − 0.54 MPa for germination. In conclusion, the use of the HTT modeling approach is helpful for predicting the emergence response of parthenium in a changing climate and ultimately supportive in time scheduling of parthenium weed management in cropping systems.

## Introduction

Parthenium weed (*Parthenium hysterophorus* L.) is an invasive weed species, having a serious threat to agricultural productivity, ecosystem functioning, and health hazards throughout the world (Nguyen et al., 2017; Bajwa et al., 2018a,b). This weed is spreading in various countries of Asia (Shabbir and Bajwa, 2006; Barman et al., 2014) and threatening the native flora in a wide range of habitats and agro-climatic regions (Oudhia, 2000; Matzrafi et al., 2021). Drought tolerance, high fecundity (2,000 seeds/plant), allelopathic properties, and efficient wind dispersal enable the stable existence of parthenium under harsh environmental conditions (Bajwa et al., 2018a; Matzrafi et al., 2021). Moreover, each plant has the capability to produce an extensive seed bank very quickly, which enhances its survival rate (Adkins and Shabbir, 2014; Matzrafi et al., 2021). Parthenium also had a tendency to long-term persistence in different ecological conditions and different habitats (Navie et al., 1998).

In the life history of a plant, both seed germination and seedling emergence are important basic processes responsible for the success and existence of adult individuals (Del Monte and Dorado, 2011). Both processes are largely controlled by temperature (Jones et al., 2013) and water potential (Bello and Bradford, 2016). Seed germination is a widely documented natural phenomenon, which displays a diversity among different plant species as each species has a characteristic germination season (Baskin and Baskin, 2014). The germination response of weed seeds may vary considerably under a wide range of temperatures (Adegbaju et al., 2018). The capability to forecast weed germination and emergence under seasonal temperature and moisture regimes might help to enhance crop management by enabling the application of highly effective weed management and control strategies by optimizing the time schedule (Leblanc et al., 2004; Myers et al., 2004). Both soil temperature and water potential play roles as key factors in determining the rate of germination (GR) and emergence, and these phenomena can be predicted by using the hydrothermal time (HTT) modeling approach (Bair et al., 2006; Haji and Gonzalezandujar, 2009).

The germination pattern of a seed sample can be well defined and predicted through population-based models under a wide range of conditions (Bello and Bradford, 2016; Bradford, 2018). Such models are commonly called population-based threshold (PBT) models, particularly the thermal time (TT), hydrotime (HT), and HTT models, respectively, which represent the effect of temperature, water potential, and their interaction (Garcia-Huidobro et al., 1982; Gummerson, 1986; Bradford, 1990; Alvarado and Bradford, 2002; Batlla and Benech-Arnold, 2015; Donohue et al., 2015; Bello and Bradford, 2016). These models are successfully used for the prediction of germination response of various crops (Finch-Savage and Phelps, 1993; Alvarado and Bradford, 2002) and weedy species (Huo et al., 2016) and are helpful for weed management. In this way, weed control measures are becoming progressively applicable by the farming community because of reducing the pressure of herbicide input and supporting the adaptation of non-chemical weed control methods (Grundy et al., 2000).

Population-based threshold models were developed on the basis of physiological processes, which occur during germination under the influence of environmental factors (Allen et al., 2000). The role of temperature is considered a major determinant regulator for controlling germination because it provides the accumulation of heat units within the range of cardinal temperature (Bradford, 2002; Reed et al., 2022). Seed germination becomes speedy with increasing temperature from base temperature to an optimum level and then starts to reduce with increasing temperature from optimum level to the highest level (ceiling temperature) (Steinmaus et al., 2000; Bradford, 2002; Rows and Finch-Savage, 2003). These cardinal temperatures are fully related to geographical and ecological situations as germination and growth behavior of a species totally depend on temperature range, which controls the subsequent growth and development of a plant (Baskin and Baskin, 2014).

Seeds germination is subjected to water availability (Reed et al., 2022). For this purpose, the HT model is used for quantifying seed germination under different levels of water potential (Bradford, 1990; Batlla and Benech-Arnold, 2015; Daibes and Cardoso, 2020). The HT concept is similar to the TT model, which is used to describe the germination time and rate on a quantification basis, and this model works in response to different levels of water potential in the germination medium.

It would be helpful for timely weed management of parthenium through accurate predictions of germination of this weed. Limited research has been reported on the prediction of parthenium through the use of PBT models. Therefore, the present study focuses on the application of PBT models for predicting and estimating the germination and emergence pattern of parthenium seeds at varying temperature and osmotic stress conditions. The objectives of the study were (1) to quantify the germination response of parthenium seeds to temperature and osmotic stress and (2) to explore the germination behavior of parthenium seeds by using the concept of germination model, i.e., TT, HT, and HTT, and also to provide the data set of germination thresholds and parameters, which will be helpful for the timing of weed control.

## Materials and methods

### Crop husbandry and treatments

Fresh achenes were collected from adult plants grown in the areas of Ayub Agricultural Research Institute (AARI) (31°41 N 73° 12 E) and the University of Agriculture, Faisalabad (31.42° N, 73.07° E), Pakistan between November 2018 and December 2018. Seeds were sun dried and cleaned manually on the threshing floor of Plant Physiology Research Area, AARI, Faisalabad, Pakistan. Initial seed quality was determined according to the International Seed Testing Association (ISTA) rules before conducting the lab experiments (International seed testing association [ISTA], 2021).

Seed germination tests were conducted in Petri dishes having two layers of moistened blotting papers in a locally made thermogradient table (International seed testing association [ISTA], 2021). The thermogradient table consisted of an aluminum plate with having a size of 75 × 75 × 3.5 cm with four partitions. Different temperatures were maintained under three chambers at a time, as cool water and hot water are pumped through the channels with the help of two circulating water baths that were used to create a temperature gradient. The thermal conditions on the gradient table were validated by keeping the thermometers in different chambers. About 25 seeds per Petri dish with ten replications for each treatment (250 seeds per treatment) were used and exposed to different constant temperatures ranging from 5 to 40°C. Similarly, parthenium seeds were also exposed to different water potentials ranging from 0 to −1.0 MPa under three temperatures (10, 20, and 30°C). Each Petri dish was counted as a replication. Osmotic solutions of different water potentials (0, −0.25, −0.50, −0.75, and −1.0 MPa) were prepared by using polyethylene glycol (PEG-8000). PEG was dissolved in distilled water to make different required osmotic solutions according to Michel (1983) and verified by using an osmometer (Wescor, United States). To avoid changes in the water potential of the germination media, the solutions were substituted by new ones every 24–48 h. Germinated seeds were counted two times per day for the period of 10 days. Seeds were considered as geminated upon the emergence of a visible radicle with a length of 2 mm.

After recording data of daily germination count, final germination, time taken to 50% germination, and mean germination time under the effect of temperature and water potential were measured according to the equations of Ellis and Roberts (1981) and Coolbear et al. (1984). Cumulative germination percentage was transformed to probit and regressed against the time log (Finney, 1971; Steinmaus et al., 2000), and germination time (t_g_) was taken according to Steinmaus et al. (2000). Similarly, GR was calculated through the inverse of germination times for every percentile under each temperature and water potential. The estimated parameter values under TT and hydrothermal models were achieved through plotting germination rate vs. temperature and water potential for each percentile. Then repeated probit analysis was used for calculating the exact parameters of TT, HT, and HTT for the whole population.

The rates of germination were separated into suboptimal and supraoptimal ranges. TT models were applied for the quantification of germination rate under the effect of different temperatures (heat units) (Bradford, 2002). From the germination data, the values of base temperature and ceiling temperature were predicted (Ellis et al., 1986). According to this model, the required TT to germination fraction at suboptimal and supraoptimal can be calculated for germination “g,” respectively;


$$\theta_{T_{1}{(}_{g})}=\left(T-T_{b}\right)t_{(}{}_{g)}$$

And,


$$\theta_{T_{2}{(}_{g})}=\left(T_{c}\left(g\right)-T\right)t_{(}{}_{g)}$$

where T, T_b_, and T_c_ are the actual, base, and ceiling temperatures, respectively, and t(g) shows the time for germination of g. TT can be determined by repeated probit regression analysis (Boddy et al., 2012).


Probit(g)=[(T-Tb)t(g)-θT(]50)/σθT

From the above equation, probit (g) is the probit transformation about the cumulative germination percentage “g.” θ_*T*_(50) and σθ_T_ are the median TT or TT to 50% germination and standard deviation (SD) of θ_T_ among the individual seeds for the seed population. When T_b_ is predictable, TT for germination t(g) can be normalized *via* multiplication of the factor (T − T_b_).

Germination response with respect to water potential (ψ) is described by the HT model. HT (MPa-day or hour) was proposed by Gummerson (1986) and Bradford (1990) for explaining the germination behavior under the effect of water potential. The HT constant, θ_H_, is determined as:


θH=(ψ-ψb(g))tg

where ψ is used for water potential, and ψb(g) is for base water potential. For estimating the parameters in HT for the whole seed populations, a repeated probit regression analysis can be used to determine HT constant (Bradford, 1990) as:


Probit(g)=[ψ-(θH/tg)-ψ]b(50)/σψb

where ψ_b(50)_ is the median of ψb, and σψ_b_ is the SD of ψ_b_ among different seeds within a seed population.

When TT and HT models have been combined, then a HTT model was developed and used for quantifying seed germination under the combined effect of temperature and water potential (Alvarado and Bradford, 2002; Rows and Finch-Savage, 2003). According to the HTT model, the seed germination time-course for suboptimal and supraoptimal temperatures can be calculated, respectively by:


θ=H⁢T(Ψ-Ψ)b(T-T)btg

and


θ=H⁢T[(Ψ-Ψ-b-(K(T-T)oT)](T-T)btg

Here θ_HT_ is the HTT constant (MPa°C days), which covers the constant for the whole population, To is the estimated value of optimum temperature at which maximum germination was occurred and K_T_ is a constant when T > To (Alvarado and Bradford, 2002).

The parameters related to HTT models can be estimated when repeated probit analyses are used by the following equations;


Probit(g)=[Ψ-(θ/H⁢T(T-T)bt-gΨ(50)b]/σψb

Probit(g)=[Ψ-(K(T-T)°T)-(θ/H⁢T(T-T)bt-gΨ]b(50)/σψb

Here on probit scale, germination percentages at suboptimal and supraoptimal temperatures ranges were regressed as Ψ - θ_HT_/(T − T_b_) t_g_ and Ψ- (K_T_(T − T_°_)) - (θ_HT_/(T − T_b_) t_g_, respectively. Similarly, the values, such as θ_HT_, T_b_, T_o_, and K_T_, were achieved till best values (Bradford, 1995, 2002).

### Statistical analysis

The means of different treatments were compared using the least significance difference (LSD) test at a 5% probability level (*p* ≤ 0.05) with a statistical software package “Statistix 8.1”.^1^

## Results and discussion

The principal environmental sensor curbing germination is temperature. Temperature seems to have two separate consequences, the first responds to dormancy itself and the second identifies the pace of development to germination through non-dormant seeds. This has long been acknowledged that lower limit (Tb), optimized (To), and peak (Tc) temperatures govern the germination of seeds. The decrease in germination rates and percentages occurs when T > To is not predicted by the thermal model. Empirical models that can resemble the subtle shifts in germination in this range of temperature have already been proposed. The HT and HTT models reveal how physiological and environmental variables are related to oversee seed population germination behavior.

### Thermal time analysis

**FIGURE 1:**
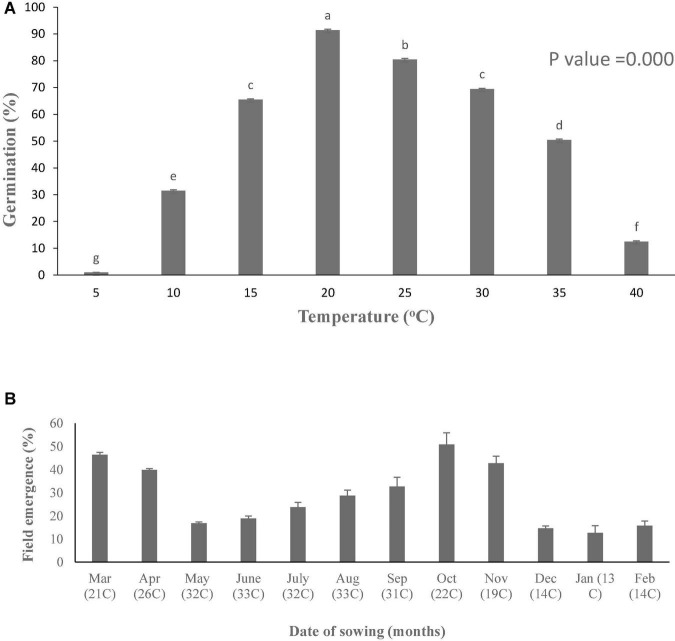
Germination potential **(A)** at different constant temperatures under lab and field emergence **(B)** of *Parthenium hysterophorus* during years 2019–20. Vertical bars showing different letters are significantly different. Values are means ± standard error.

In the present study, germination pattern of parthenium seeds was different at various constant temperatures, i.e., low and high temperatures (Figure 1). Germination rate (GRg) of parthenium weed was plotted against temperature, and germination was linearly increased toward optimum temperature and decreased with increasing temperature toward ceiling temperature. Germination response of parthenium with respect to temperature was well described with the help of TT model using suboptimal and supraoptimal range of temperature at the control level of water potential (0 MPa). According to this model, the base temperature was estimated as 7.2°C under suboptimal temperature and the temperature of 42.8°C was referred as ceiling temperature at supraoptimal temperature (Table 1). Similarly, the optimum temperature for this worst weed was estimated at 20°C from both suboptimal and supraoptimal temperature ranges.

**TABLE 1:** Model parameters for thermal time model describing germination of *Parthenium hysterophorus* seeds at sub- and supraoptimal temperatures.

Temperature (°C)	θ_T(50)_ (°C h)	T_b(50)_/T_c(50)_ (°C)	σ_Tb_ (°C)	*R* ^2^
Sub optimal (5–20)	3.01	7.2	0.30	0.957
Supra optimal (20–40)	3.21	42.8	0.37	0.970

**FIGURE 2:**
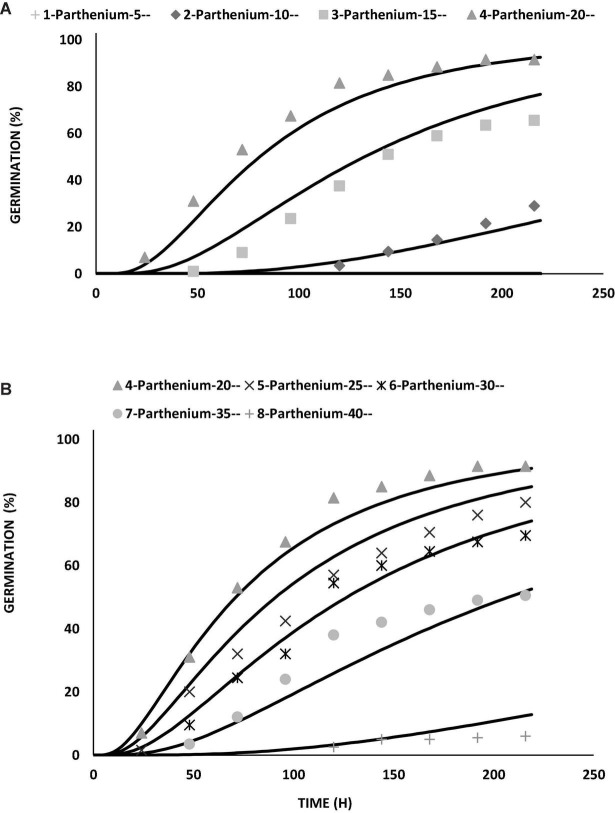
Germination curve for *Parthenium hysterophorus* seeds at suboptimal **(A)** and supraoptimal **(B)** temperatures by using thermal time modeling approach under controlled environment (lab conditions).

The value of thermal constant (θ_2_) at supraoptimal temperature is high as compared to suboptimal temperature that gives indication of more germination rate at high temperature as compared to low temperature (Figure 2 and Supplementary Figure 1). As temperature decreased, there was an increase in the number of days taken to germinate but the TT accumulation was constant. On the base of degree hours, it is easy to forecast the germination pattern of parthenium under cold and hot weather conditions. The different values of growing degree hours at different temperatures are also an incentive for predicting the time of weed management through how much percentage of parthenium seeds would be able to germinate under different climatic conditions (Haji and Gonzalezandujar, 2009). These variations were occurred due to physiological changes in seeds during germination in changing temperatures (Jones et al., 2013; Correa et al., 2020) and these fluctuations were proved to be an imperative signal toward the germination and emergence pattern of parthenium in the field and ultimately toward the control strategies of parthenium weed at the proper time.

### Hydrotime analysis

**FIGURE 3:**
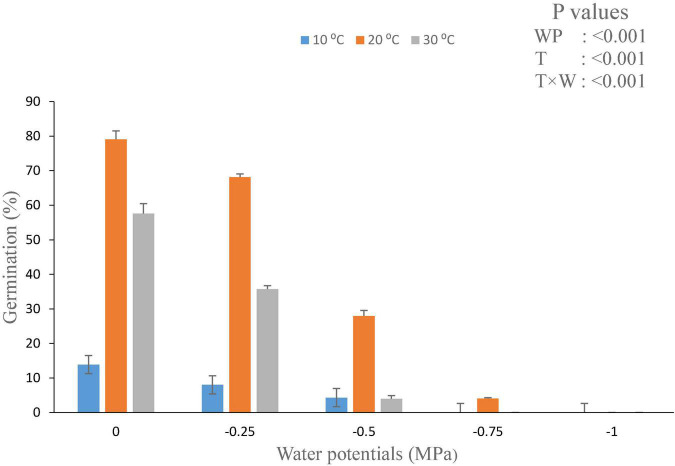
Interactive effect of water potential and temperature on germination of *Parthenium hysterophorus* seed under controlled conditions. Values are means ± standard error.

**FIGURE 4:**
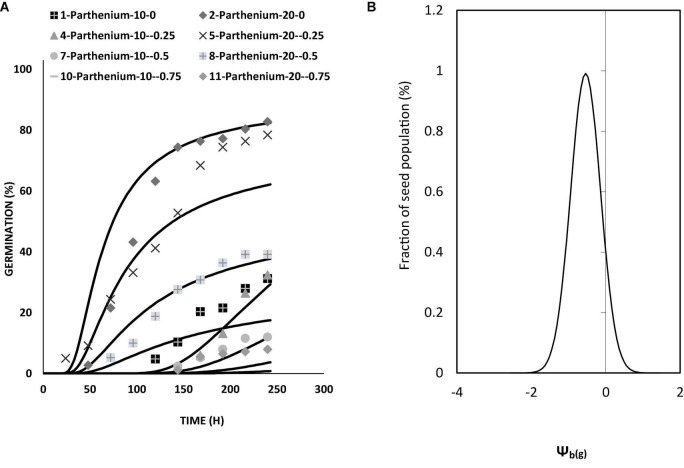
**(A)** Germination curve of *Parthenium hysterophorus* at different levels of water potential and temperatures by using the hydrothermal time model under controlled environment (lab condition). **(B)** Model distribution for germination of parthenium seeds showing different frequencies of Ψ_b(g)_ at different levels of water potentials and temperatures by using the hydrothermal time model under controlled environment (lab condition).

The values of θ_H_ and ψb(50) were decreased with the increasing temperature. From the model data, it was found that HT value and HT constant (θ_H_) (MPa hours°C) for all osmotic potential levels at 10°C were different (Table 2). The germination rate and germination percent of parthenium were highest at all water potentials and at a temperature of 20°C, which is supported by both measured and predicted values from the hydrothermal approach (Figures 3, 4). Thus, it shows the biological relevance of the model to describe the effects of water potential on germination (Daibes and Cardoso, 2020).

**TABLE 2:** Model parameters for the hydrotime model describing germination of *Parthenium hysterophorus* at different temperatures.

Temperature (°C)	θ_H_ (MPa h)	Ψ_b(50)_ (MPa)	σ_Ψb_ (MPa)	*R* ^2^
10	263.4	–0.77	0.85	0.884
20	65.0	–0.70	0.35	0.942
30	35.8	–0.20	0.42	0.918

Hydrotime constant value (35.8) at warm temperature is less as compared to the value calculated at optimum temperature (20°C) and low temperature (10°C; Table 2). Our findings suggest that parthenium does have a remarkable ability to germinate at reduced moisture levels, which is compatible with its high invasiveness and drought tolerance throughout its life span. At a typical day/night temperature of 27°C, Tamado et al. (2002) found that germination was completely inhibited above − 0.52 MPa osmotic potential. Interestingly, even at − 0.86 MPa and a lower mean day/night temperature of 20°C, the same study found that around 20% of seeds were germinated (Bajwa et al., 2018b).

### Hydrothermal time analysis

The hydrothermal time model exhibits that both temperature and water potential had a significant effect on the germination rate and germination percentile of parthenium seeds. From the model perspective, maximum germination of parthenium seeds was occurred at 20°C by using water potential of − 0.25 MPa (Figure 3 and Supplementary Figure 2). Germination of parthenium was decreased as water potential was increased toward base water potential at low and high temperatures.

**FIGURE 5:**
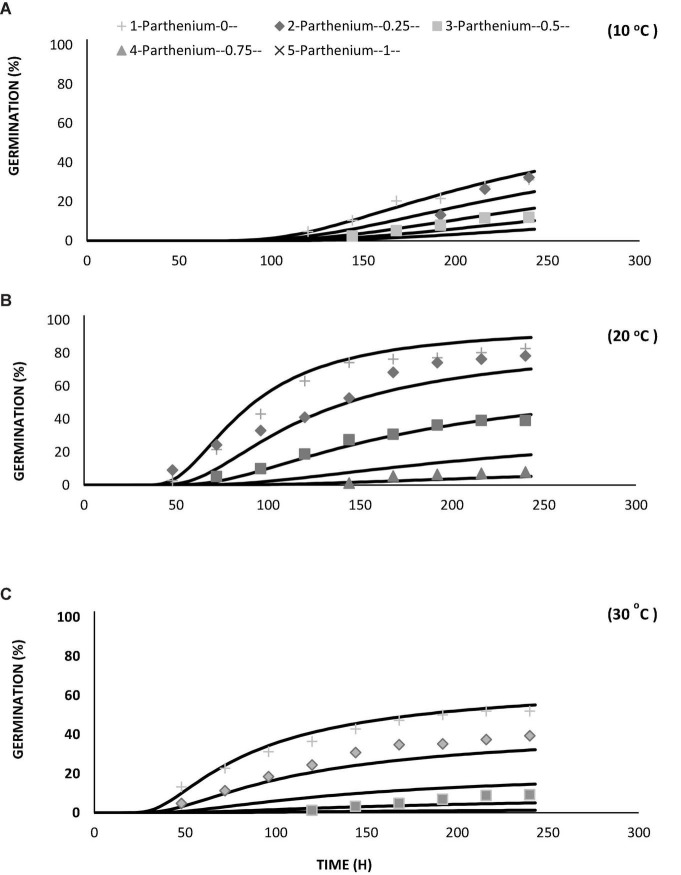
Germination curve of *Parthenium hysterophorus* at different levels of water potentials by using hydrotime model under controlled environment at temperatures of **(A)** 10, **(B)** 20, and **(C)** 30°C.

Germination percentage and germination time were lowered and increased at low and high temperatures, respectively. At the ideal temperature and water potential conditions, fewer MPa°C hours were needed for germination (Figure 5). Parthenium had no germination below the base temperature (Table 3). Using responsive dynamic models for species in the same community, researchers discovered that distributing germination within and between years is generally beneficial, yet these species could use holistic strategies that combine predictive plasticity in germination fractions or timing with bet hedging (Gremer et al., 2016). In the field, early vs. late germination fractions, as well as overall germination fractions, adjust from year to year. During years when more seeds tended to germinate early in the season, early germinates tended to have greater lifetime fitness, demonstrating predictive adaptability in germination frequency in combination to bet hedging. Seeds are also inclined to germinate to larger proportions in years with good survival and fecundity (Liu et al., 2019). Generally, the ability of parthenium biotypes to germinate over a wide temperature range might allow them to overrun. Likewise, a much enhanced germination rate at higher day/night temperatures may promote its dissemination in the future as a result of climate change (Bajwa et al., 2018b; Correa et al., 2020). According to the findings of this study, a reduction in water potential led to a decline in germination rate. Wang (2005) discovered a substantial connection involving germination rate and temperature, as well as a decrease in water potential. Normally, water likened to temperature seems to have a more complex influence on germination, particularly at low water potential. Once water potential is lower under the threshold of radical upsurge, biochemical development is noticed. The vulnerability to low water seems to be under physiological regulation or could be the result of seeds’ physiological adaptation to conditions around the temperature or water threshold (Zaferanieh et al., 2020).

**TABLE 3:** Model parameters for the hydrothermal time model describing germination of *Parthenium hysterophorus* seeds under controlled environment (lab condition).

Temperatures (°C)	θ_H_ (MPa °C h)	Ψ_b(50)_ (MPa)	σ_Ψb_ (MPa)	*R* ^2^
10 and 20	516.6	–0.54	0.40	0.867

In summary, parthenium is a global invasive weed with a considerable proclivity to sprout in a range of ecological circumstances. The importance of PBT models in predicting germination and emergence of weedy plants in field environments could be significant. Such models have proven to be an efficient tool for predicting the emergence of weeds based on field conditions in a climate-change scenario. It is simple to predict the germination pattern of parthenium under cold and hot weather conditions using population-based models. According to these models, parthenium weed could germinate and emerge at quite a variety of temperatures, having T_b_ of 7.2°C and T_c_ of 42.8°C, with the highest germination at moderate temperatures (20–25°C). Likewise, this weed has the ability to germinate at various water potentials and temperatures, with more germination predicted at water potential (0 to − 0.25 MPa) and temperature of 20°C, respectively.

## Conclusion and future prospects

This argument is supported by the strong effect of variations in soil temperature and moisture across natural habitats. Furthermore, this occurrence has significant ramifications within the framework of future climate conditions since it suggests that expected temperature variations in the vicinity would have a significant impact on the time of parthenium germination in their current distribution zones. The wide range of germination temperatures of species shows that germination activity could readily adjust to climate change. This could have a big impact on anticipating future consequences of global warming on plant dispersal. The information presented here should be used in programs examining species sensitivity to climate change so that weed researchers could be able to develop effective weed management tactics for such an aggressive weed based on forecasts made using PBT models.

## Data availability statement

The original contributions presented in this study are included in the article/Supplementary material, further inquiries can be directed to the corresponding author.

## Author contributions

All authors contributed significantly in conceptualization, writing – review and editing, and funding acquisition of the current study.

## Footnotes

1
https://www.statistix.com/
