# Supplementary material for: Quantifying the germination response of Parthenium hysterophorus at various temperatures and water potentials by using population-based threshold model
Source: Front Plant Sci. 2022 Aug 10;13:961378. doi: 10.3389/fpls.2022.961378 (PMC9399799; doi:10.3389/fpls.2022.961378)
Supplement: Supplementary file 1 [file Data_Sheet_1.ZIP › Supplementary Figures.docx]

| **(a)** |
| --- |
| **θ_T(g)_** |
| **Supplementary Figure 1:** Model distribution showing the relative frequencies of θ_T(50)_ and of T_C(50)_ values at sub optimal temperature (a) and supra optimal temperature (b) under controlled environment (Lab conditions). |

| **(10 ^o^C )** |
| --- |
| **(30 ^o^C )** |
| **(30 ^o^C )**  **Ψ_b(g)_** |
| **Supplementary figure 2.** Model distribution for germination of parthenium seeds showing different frequencies of Ψ_b(g)_ at different levels of water potentials by using hydro time model under controlled environment at temperature of 10, 20 and 30 ^o^C |
